# Noninvasive Ultra Low Intensity Light Photodynamic Treatment of Glioblastoma with Drug Augmentation: LoGlo PDT Regimen

**DOI:** 10.3390/brainsci14121164

**Published:** 2024-11-21

**Authors:** Richard E. Kast, Anton P. Kast, Jürgen Arnhold, Felix Capanni, Laura N. Milla Sanabria, Nicolas Bader, Bruno Marques Vieira, Alex Alfieri, Georg Karpel-Massler, Erasmo Barros da Silva

**Affiliations:** 1 IIAIGC Study Center, 11 Arlington Ct, Burlington, VT 05408, USA; antonkast@gmail.com; 2 Institute for Medical Physics and Biophysics, University of Leipzig, Härtelstrasse 16-18, 04107 Leipzig, Germany; juergen.arnhold@medizin.uni-leipzig.de; 3 Biomechatronics Research Group, Ulm University of Applied Sciences, Albert Einstein Allee 55, 89081 Ulm, Germany; felix.capanni@thu.de (F.C.); nicolas.bader@thu.de (N.B.); 4 INBIAS, Universidad Nacional de Río Cuarto-CONICET, Río Cuarto 5800, Argentina; lmilla@exa.unrc.edu.ar; 5 Laboratório de Biomedicina do Cérebro, Instituto Estadual do Cérebro, Rio de Janeiro 20230-024, Brazil; brunomarquesv@gmail.com; 6 Department of Neurosurgery, Cantonal Hospital of Winterthur, 8400 Winterthur, Switzerland; alex.alfieri@ksw.ch; 7 Department of Neurosurgery, Ulm University Hospital, 89081 Ulm, Germany; georg.karpel@gmail.com; 8 Neurosurgery Department—Neuro-Oncology, Instituto de Neurologia de Curitiba, Rua Jeremias Maciel Perretto, 300-Campo Comprido, Curitiba 81210-310, Brazil; erasmo-inc@uol.com.br

**Keywords:** 5-ALA, glioblastoma, noninvasive, photodynamic treatment, repurposing

## Abstract

This paper presents the basis for LoGlo PDT, a new treatment for glioblastoma. Glioblastoma is currently treated with maximal safe resection, temozolomide, and ionizing irradiation. Mortality in 2024 remains over 80% within several years from diagnosis. Oral 5-aminolevulinic acid (5-ALA) is an FDA/EMA approved drug that is selectively taken up by malignant cells, including by glioblastoma. In photodynamic treatment of glioblastoma, intense intraoperative light causes glioblastoma tissue that has taken up 5-ALA to generate cytotoxic reactive oxygen species. The requirement for intense light flux has restricted photodynamic treatment to a single one-hour intraoperative session. We analyze here published data showing that external light, illuminating the entire intact scalp, can attain low μW/cm^2^ flux several cm into intact brain that would be sufficient to mediate 5-ALA photodynamic treatment of glioblastoma if the light and 5-ALA are delivered continuously over 24 h. At the core of LoGlo PDT regimen is the dataset showing that, for a given fluence, as the duration of PDT light delivery goes down, light intensity (flux) delivered must go up to achieve the same glioblastoma cell cytotoxicity as would a weaker light (lower flux) delivered over a longer time. Thus, a repetitive, noninvasive PDT of glioblastoma using an external light source may be possible. We analyze 5-ALA cellular physiology to show that three non-oncology drugs, ciprofloxacin, deferiprone, and telmisartan, can be repurposed to increase light energy capture after 5-ALA, thereby increasing photodynamic treatment’s glioblastoma cell cytotoxicity. The LoGlo PDT approach uses both drug augmentation and prolonged ultra-low noninvasive transcranial light delivery for a repetitive, noninvasive 5-ALA photodynamic treatment of glioblastoma.

## 1. Introduction

This paper presents details on a new, low risk treatment for glioblastoma (GB). The new treatment, termed LoGlo PDT, resulted from our analysis of current photodynamic treatment (PDT), the cellular physiology of GB, and the light transmission and diffusion characteristics of human skull and brain tissue.

We conclude that low-intensity transcranial light can be noninvasively and repeatedly delivered to mediate effective PDT if delivered over 24 h. Furthermore, we show how three drugs from general medical practice can be repurposed to make photodynamic treatment more effective. LoGlo PDT combines using low wattage transcranial light to the scalp surface with oral 5-aminolevulinic acid (5-ALA) and drug augmentation to create a method for a repeated, noninvasive photodynamic treatment of GB that is available today, with today’s medicines, and with today’s readily available commercial light sources.

Ten years ago, GB’s fatal recurrence rate was almost 100% [1]. Despite improvements in the interval’s standard of care, the same can be said today.

## 2. 5-ALA

GB cells tend to take up 5-ALA with greater avidity than other brain cells [2,3,4]. This has two important consequences for GB treatment:

- 5-ALA allows intraoperative PDT;
- A fluorescent metabolic product of 5-ALA, protoporphyrin IX (PpIX), allows a more complete primary tumor resection, termed fluorescence guided resection.

In intraoperative 5-ALA PDT, after 5-ALA uptake by GB cells, 5-ALA is metabolized to PpIX. Intraoperative bright light exposure results in light energy being transduced by intracellular PpIX to molecular oxygen, O_2_, creating reactive oxygen species (ROS) such as superoxide anion radical (O_2_^−^), hydrogen peroxide (H_2_O_2_), and most of all singlet oxygen (^1^O_2_). These ROS damage vital GB cell structures, resulting in cell growth cessation or cell death [4,5,6,7]. Intraoperative 5-ALA PDT prolongs GB’s progression free survival and may also prolong overall survival [6,7,8]. See Table 1 for some core pharmacological parameters of 5-ALA.

PpIX fluoresces red after excitation at 415 nm. Thus, GB tissue appears red after intraoperative illumination with 415 nm light, allowing more complete recognition and removal of GB tissue [9,10,11,12]. Fluorescence guided resection also prolongs GB’s progression free time and may prolong median overall survival. Intraoperative 5-ALA PDT and 5-ALA fluorescence guided resection can be combined with added benefit [13,14].

**Table 1:** Some core pharmacologic parameters of 5-ALA. References [15,16,17,18,19,20,21].

PpIX Fluorescence	5-ALA	LFT Elevation	MAP Decrease	Nausea
T1/2 = 10 min *	T1/2 = 1–3 h	4%	11%	15%

MAP, mean arterial pressure decrease > 20 mm Hg; LFT, liver transaminases; * Clinically, fluorescence and PpIX peak 7–8 h following oral administration of 5-ALA.

A second dataset, detailed below, analyzes peer reviewed data on three generic drugs from general medical practice showing that they have potential for repurposing to enhance 5-ALA PDT cytotoxicity to GB cells. These drugs are the antibiotic ciprofloxacin, the iron chelator deferiprone, and the hypertension treatment drug telmisartan. LoGlo PDT uses these repurposed drugs and long duration, noninvasive delivery of transcranial light for ultra-low flux, drug augmented, 5-ALA PDT.

## 3. Ultra-Low Flux PDT

Current intraoperative 5-ALA PDT consists of giving oral 5-ALA before surgery and then, after resection, delivering 635 nm light, ~200 J/cm^2^ total, to the resection cavity wall in five fractions of 12 min each with 2 min pause between the four periods. There are multiple variations on this basic regimen [2,3,4,5,6,8,10,13]. See Table 2 for fluence and flux definitions.

LoGlo PDT is an extension of past studies using low-flux 5-ALA PDT in the range of μW/cm^2^, roughly < 50 J/cm^2^ total, delivered over many hours [22,23,24,25,26,27,28,29]. The flux necessary for tumor cell cytotoxicity goes up as the time over which it is delivered goes down. Already in 1998, there were data indicating that lower total fluence was needed for the same cytotoxicity if lower fluxes were used over longer times [26].

The problems of current PDT for GB that LoGlo addresses can be summarized as follows:

- We know that at the time of diagnosis, GB cells reside throughout the entire brain. GB is a whole brain disease. Intraoperative PDT is only effective within the first 1 or 2 cm within the resection cavity wall.
- PDT is restricted to a single intraoperative session.
- Rapid oxygen depletion with high-flux PDT limits effectiveness. Ultra-low flux, prolonged duration PDT has demonstrated lower oxygen consumption per second in experimental murine xenograft models [22,23,25].
- Some GB cells contain inadequate PpIX for effective PDT cell killing [30,31]. The three adjunctive repurposed medicines of LoGlo PDT are meant to at least partially redress that.

By leaving the resection cavity open for several days, repeating PDT would be possible, but constrained by infection risk and procedure complications. A fully implantable light and power source, the Globus Lucidus [32], is in development to allow long-term, repeated closed PDT delivered to the resection cavity wall. Leaving transcranial fiberoptic light guides in place to deliver PDT using an external light source, termed interstitial PDT, is being explored to allow several post-resection PDT sessions, but it has not shown great effectiveness and carries infection risks associated with leaving skull-penetrating external light conduits in place [33,34,35,36].

Morse et al. showed that an optical window above 650 nm and below 1000 nm allowed useful 940 nm light energy to penetrate 4 cm into the human unfixed cadaver head [37]. Morse et al., using a 4 watt, 940 nm light external to human cadavers’ skull, found that the degree of brain penetration was highly variable between locations along the scalp and between the cadavers tested [37]. Morse et al.’s data, below, are from four cadavers:


**Locus of Applied **

**External 4 W Light**

**Range of Light Reaching 4 cm**

**Deep to Skull in 4 Cadavers**
frontal32 to 278 μW/cm^2^parietal134 to 395 μW/cm^2^temporal7 to 234 μW/cm^2^occipital1 to 56 μW/cm^2^

The wide range of penetrations means that these are only approximate indicators of the ranges we might expect clinically. Also, different wavelengths will have different μW/cm^2^ penetration values.

Mathews et al. [38] and others [22,23,24,25,26,27,28,29] have explored preclinical ultra-low flux (<50 μW/cm^2^) 5-ALA PDT, showing that it can effectively kill or stop growth of malignant cells if the excitation light is delivered continuously over longer exposure times and repeated. Already thirty years ago, it was observed that there was “significantly greater delay in the growth of tumors exposed to 50 mW/cm^2^/2 h continuously, compared to controls or to tumors exposed to the same total fluence but with light delivered at 100 or 200 mW/cm^2^.” [29]. This is the core finding prompting development of LoGlo PDT.

Key points from Matthews et al.’s study [38]:

- Repeated ultra-low flux on the order of 17 µW/cm^2^ over 24 h (1.5 J/cm^2^) is more effective in killing GB cells than the same or even greater fluence from mW/cm^2^ flux delivered over a shorter time [22,23,24,25,26,27,28,29,38].
- Repetitive treatments enhance effectiveness.

Our interpretation of the work of Tedford et al. [39], Morse et al. [37], and Mathews et al. [38] is that 10 to 20 μW/cm^2^ flux can be noninvasively delivered to the human brain to a depth up to 5 cm using a tolerable 635 nm surface light source. For comparison, note that midday sunlight shining on the bare human head delivers approximately 130 mW/cm^2^ broad spectrum light.

Thus, LoGlo PDT treats GB with broad head illumination by an external multi-LED cap after oral 5-ALA to deliver 24 h continuous low μW/cm^2^ PDT.

## 4. Safety of 635 nm Light

A procedure called photobiomodulation uses external, transcranial light with the aim of treating a variety of non-malignant brain function deficits. Wavelengths ranging from 610 to 950 nm, fluxes ranging from 2 mW/cm^2^ to 350 mW/cm^2^, and fluences from 1 J/cm^2^ to 42 J/cm^2^ using 10 to 40 W external light sources have a well-established history of safe use in humans [40,41,42,43,44,45,46,47,48].

Dozens of clinical studies in humans have explored red (~630 nm) to near infrared (~1000 nm) photobiomodulation to reduce impairments of Alzheimer’s disease, cognitive or executive function deficits, Parkinson’s disease, traumatic brain injury, autism, and other brain function conditions [40,41,42,43,44,45,46,47,48]. The results of these studies have not always shown benefit, although some have, but they all have shown good safety with minimal or no side effects from this light delivery.

A wide variety of these photobiomodulation helmets delivering broad head illumination at 600 nm to 1000 nm are commercially available for home use without prescription [49].

**Figure 1:**
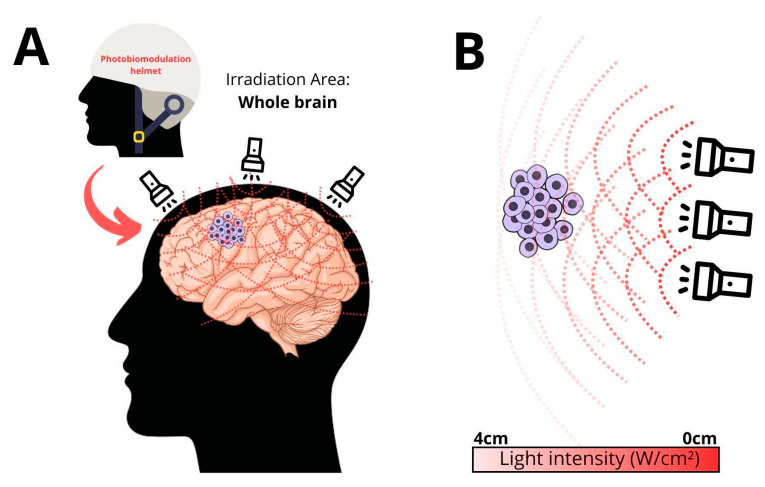
(**A**) shows how a cap with 80 LEDs will illuminate the entire brain. (**B**) indicates how diffusion of any beam of light by brain tissue can work in our favor as well as being a drawback. Proximal LEDs’ light diffuses away from our target GB area, but this light will be, to some unknown degree, compensated for by light from further away, off center, LEDs’ light diffusing toward our target area.

Figure 1 illustrates our planned transcranial LED illumination of GB. The schematic indicates rapid light diffusion by brain tissue resulting in light hitting the GB resection area from many angles. LoGlo PDT light delivery will be from 80 LEDs evenly spaced over the entire scalp area. For clarity, only three LEDs are shown.

As an example of 5-ALA’s safety, using 60 mg/kg oral 5-ALA followed by 100 J/cm^2^ delivered over 10 min to unresectable esophageal cancer resulted in improvement of dysphagia and no increase in hepatic transaminases (alanine transaminase, aspartate transaminase, alkaline phosphatase, and total bilirubin) [50]. In GB patients with preexisting transaminase elevation, 30% experienced further increases after 5-ALA 20 mg/kg assisted resection. The elevations were of grade 1, 2, or 3 and were temporary, resolving without sequelae [51]. 5-ALA fluorescence guided GB surgery in those with preoperative normal LFTs has a similar percent benign, rapidly resolving, low post-operative transaminase elevations [52,53].

## 5. The LoGlo PDT Drugs

The second part of LoGlo PDT is the addition of three already marketed drugs, repurposed to enhance 5-ALA PDT effectiveness. Drug repurposing refers to the use of previously approved drugs that induce basic physiology changes that are beneficial in treating conditions other than their traditional or originally approved use. Table 3 lists the three drugs of LoGlo with their common general medicine use and their repurposed use during 5-ALA PDT.

Two of the three drugs discussed here, ciprofloxacin and deferiprone, were discussed in a previously published paper as potential adjuncts to PDT in GB [54]. Ciprofloxacin and telmisartan have a database showing GB growth inhibition independent of any effects on PDT or 5-ALA. Table 4 lists some basic pharmacologic parameters for the three LoGlo PDT augmentation drugs.

**Figure 2:**
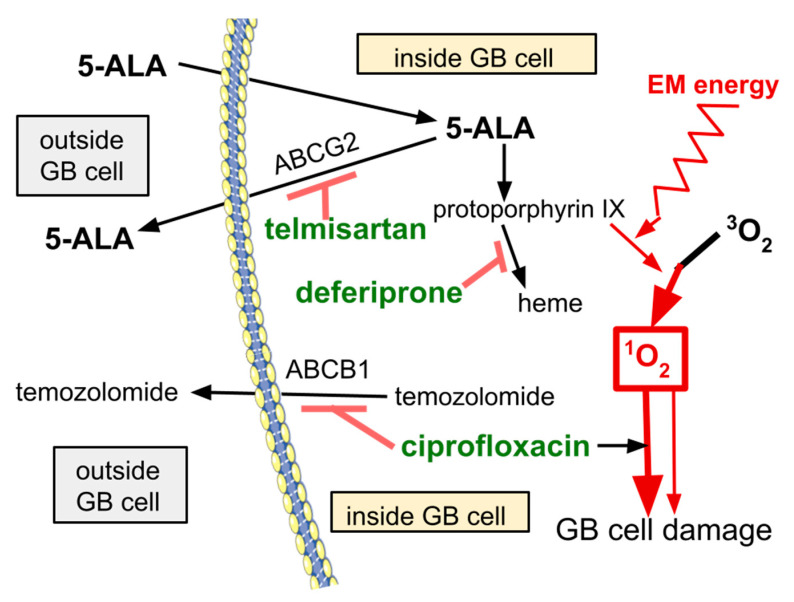
Depicts 5-ALA entry into GB cells and its indirect action in creating cytotoxic ROS after cells’ exposure to an electromagnetic field (light). By inhibiting 5-ALA export, telmisartan increases intracellular 5-ALA. By inhibiting diversion of PpIX to heme synthesis, deferiprone increases intracellular PpIX. ABCG2 drug efflux pump, synonymous with BRCP; ABCB1, a drug efflux pump.

Figure 2 is a schematic drawing of the intended action of the LoGlo drugs. Data supporting the intended action are detailed in the specific drug’s section below.

## 6. Ciprofloxacin

Ciprofloxacin is a broad spectrum antibiotic in common use in humans around the world. As an antibiotic, ciprofloxacin works by inhibiting bacterial DNA gyrase while also stabilizing DNA strand damage created by DNA gyrase and topoisomerase IV. Repositioning ciprofloxacin as a cancer cell cytotoxic drug has a mainly empirically based research database rationale. The mechanism by which it exerts malignant cell growth inhibition has not yet been established [58,59,60,61,62].

The primary reason for ciprofloxacin’s inclusion in LoGlo PDT was the past simple demonstration of increased in vitro PDT effectiveness in the presence of ciprofloxacin in glioma cells [63], chordoma cells [64], and meningioma cells [65].

The secondary reason for ciprofloxacin’s inclusion in LoGlo PDT was its inhibition of the cell efflux pump ABCB1, the primary export pump of temozolomide. ABCB1 knockdown increases GB cytotoxicity of temozolomide and increases brain tissue levels of temozolomide [66,67]. Conversely, enhancing ABCB1 function decreases GB cell sensitivity to temozolomide [68]. Ciprofloxacin increased intracellular levels of ABCB1 substrate drugs other than temozolomide [69].

In an in vitro model, cytotoxicity of Caesium 137 gamma irradiation of glioma cells, 0.66 MeV, 1 Gy/min, 8 Gy total, increased by adding 5-ALA. Adding ciprofloxacin to 5-ALA and that irradiation yet further increased cytotoxicity. The effect was not large, but it was clear and statistically significant [70].

Recognizing the preclinical database of ciprofloxacin’s inherent cytotoxicity to malignant cells, Gera et al. gave 1000 mg/day ciprofloxacin along with traditional chemotherapy with etoposide in acute myeloid leukemia cases in a phase 1b trial. That trial indicated a benefit from adding ciprofloxacin [71].

## 7. Deferiprone

Deferiprone is an iron binding drug in human use to reduce iron overload. In preclinical study, a series of FDA/EMA approved, iron binding drugs similar to deferiprone augmented 5-ALA cytotoxicity to GB. By binding iron, iron becomes less available for incorporation into PpIX to create heme. That metabolic impediment results in a backup of intracellular PpIX [72,73,74,75,76]. This process can be intuitively appreciated in Figure 2; as the left hand arrow going off from PpIX decreases, more PpIX is available to the right hand arrow’s process going off to singlet oxygen. [77,78,79].

Among the several FDA/EMA approved iron chelators for human use, deferiprone would be best for use in GB 5-ALA PDT because it achieves good brain tissue levels [77,78] while the other approved iron chelators do not. Deferiprone has been in use for 40 years and has a good history of tolerability, safety, and reduction in brain iron content [80,81].

## 8. Telmisartan

Telmisartan is an angiotensin II receptor 1 blocking drug (an ARB) used to lower high blood pressure. Several physiologic effects of telmisartan recommend its use during PDT treatment of glioblastoma.

Telmisartan is one of several marketed drugs that also inhibit ABCG2 [82,83,84,85]. ABCG2, also known as BCRP, is a 144 kDa homodimer efflux pump that is responsible for 5-ALA cellular export. Note that ABCG2 also promotes angiogenesis and antioxidant responses. Lower ABCG2 function, either pharmacologically induced or otherwise diminished, increases PpIX build up [86,87]. That is reflected by the increased 5-ALA PDT effectiveness and stronger PpIX mediated fluorescence guided GB visualization as ABCG2 function decreases [88,89,90,91].

Other pharmacologic effects of telmisartan have potential benefits during GB treatment. Telmisartan exerts anti-inflammatory effects by virtue of its inhibition of PPAR-gamma [92,93,94,95]. An inhibiting effect of telmisartan in several cancer types has been ascribed to several of the above mechanisms as well as empirically demonstrated [96,97,98,99,100,101]. Also, specifically in GB, telmisartan inhibits in vitro growth [102,103,104,105].

## 9. LoGlo PDT and GB Ecosystems

As with most ecosystems in nature, different interacting communities exist within a GB. These cell communities are connected by pairwise and higher order set interactions leading to a mutually supporting interdependence of the different cell communities. These ecological niches, communities of mutually supporting cells within an individual tumor, have differing radioresistances, drug sensitivities, and contain different trophic, nonmalignant cell populations. Each community has a slightly different spectrum of vulnerabilities. We reason that the broader the range of these ecosystems we can inhibit or kill, the more effective the treatment will be [106,107,108,109]. Repeated LoGlo PDT during standard temozolomide chemoirradiation aims at broadening the range of ecosystems we inhibit.

Recurrence after resection and standard temozolomide chemoradiation tends to arise from small numbers of relatively treatment resistant stem cells. Those stem cells sit at the apex of an entropic hierarchy, imparting therapy resistance [4,110,111,112]. GB stem cell subpopulations are more resistant to 5-ALA PDT than are non-stem populations [113,114]. Although GB stem cells are more resistant, they also synthesize PpIX and can be killed by 5-ALA PDT [115,116,117], highlighting the importance of defeating resistance pathways with augmentation drugs as advocated in this paper.

## 10. Limitations and Caveats

A pilot study with histologic examination of resected GB tissue after prolonged, transcutaneous ultra-low flux 5-ALA PDT with ciprofloxacin single drug augmentation would be a first step in determining the safety and effectiveness of LoGlo PDT.

There are fundamental uncertainties at several points along the proposed LoGlo PDT treatment path. We have only partial and at times contradictory data on light penetration through skin, calvaria, meninges, and brain tissue. Many interindividual differences will change the light attenuation parameters. Therefore, the light penetration values listed throughout our paper should be understood as approximate, subject to many variables for which we cannot account at the moment. Precise measurements and further study will give us a better understanding of what ultra-low flux, 24 h 5-ALA PDT can and cannot do.

The idea to use external light sources to deliver ultra-low flux at the tumor is largely based on preclinical work of Mathews et al. [38] and others [22,23,24,25,26,27,28,29], Morse et al. [37], and Tedford et al. [39]. However, preclinical results do not always transfer to clinical results.

Although preclinical studies give little grounds for safety concerns, as with any new, innovative treatment, unforeseen adverse events cannot be excluded.

Counterbalancing unforeseeable risks are as follows:

- The uniformity of fatal outcome of GB using all current treatment modalities;
- The established or predicted safety of the individual components of LoGlo PDT;
- The sound rationale predicting LoGlo effectiveness.

PpIX GB cell levels, and hence intensity of ROS generation during PDT can vary between patients and between different areas within the same tumor [118,119]. While it is hoped that drug augmentation and the longer duration of 5-ALA exposure will mitigate that heterogeneity, any PDT, including LoGlo PDT, will fail if there were insufficient GB intracellular PpIX.

Although photobiomodulation research has established the safety of human brain exposure to transcranial delivery of 635 nm light, this was established without the addition of 5-ALA and without the planned augmentation medicines. Also, photobiomodulation studies in humans, of which there are dozens, use 15 to 30 min light exposures several times a week. We are planning hours of daily light exposure, unchartered territory.

## 11. Discussion and Conclusions

The data analysis in this paper showed that low-flux light delivered to the scalp surface to effect 5-ALA PDT for GB is plausible and predicted to be benign.

Ciprofloxacin, deferiprone, and telmisartan are well-tolerated drugs in common use. Although surprises cannot be excluded, there is no a priori reason to suspect an adverse interaction when these drugs are used together.

Low flux light over longer times during 5-ALA PDT has several strong advantages over the current use of high flux, short duration intraoperative PDT:

- For the same fluence, greater cytotoxicity can be achieved using lower flux over a longer time compared to using higher flux over a shorter time [22,23,24,25,26,27,28,29].
- A given fluence delivered in many fractions is more effective than the same fluence delivered as one continuous dose [28,80,120].
- Low flux transcranial light can be repeatedly and noninvasively delivered sufficient for an effective 24 h PDT.
- Paucicellular colonies of GB anywhere in the brain have potential to be treated by LoGlo PDT.
- High flux PDT as currently practiced quickly expends exposed tissue’s oxygen. Ultra-low flux PDT as we intend may allow better continuous oxygen replenishment [22,23,25,121].

LoGlo regimen as presented here has a strong, well supported rationale. The extensive body of past research discussed in this paper indicates that the risks and side effects of LoGlo are manageable. As of 2024, GB growth and recurrence are not manageable. Therefore, a pilot study is warranted.

## Figures and Tables

**Table 2:** Fluence and flux definitions.

Term	Definition	Units
fluence	an amount, energy delivered per unit area	J/cm^2^
flux	a rate, energy delivered per unit area per second	W/cm^2^

**Table 3:** List of the augmentation drugs with their general medical use and their use in LoGlo PDT. ABCG2, a cell efflux pump, synonymous with BRCP; ARB, angiotensin 2 receptor 1 inhibitor; PpIX, protoporphyrin IX.

Drug	Common Use in General Medicine	Repurposed Use in LoGlo PDT
ciprofloxacin	as antibiotic	increased PpIX levels
deferiprone	for iron chelation	for lowering intracellular iron
telmisartan	to treat hypertension	to inhibit ABCG2, to agonize PPAR gamma, to inhibit and angiotensin receptors

**Table 4:** Several basic pharmacologic parameters of the LoGlo PDT augmentation drugs. Listed values are approximate with wide individual variations.

Drug	Oral Dose	Cmax	Tmax	T1/2	Reference
ciprofloxacin	1200 mg/d	3 µg/mL	2–3 h	4 h	[55]
deferiprone	50 mg/kg/d	42 µg/mL	30 min	2 h	[56]
telmisartan	20–80 mg/d	160 ng/mL	2 h	24 h	[57]

## Abbreviations

5-aminolevulinic acid, 5-ALA; glioblastoma, GB; photodynamic treatment, PDT; ultra-low intensity transcranial light photodynamic treatment, LoGlo PDT; protoporphyrin IX, PpIX; reactive oxygen species, ROS.
